# Osteogenesis in human periodontal ligament stem cell sheets is enhanced by the protease-activated receptor 1 (PAR_1_) in vivo

**DOI:** 10.1038/s41598-022-19520-x

**Published:** 2022-09-18

**Authors:** Tomaz Alves, Letícia M. Gasparoni, Danilo Balzarini, Emmanuel Albuquerque-Souza, Victhor de Oliveira, Emanuel S. Rovai, Jose da Silva, Aldrin Huamán-Mendoza, Luiz H. Catalani, Carla R. Sipert, Marinella Holzhausen

**Affiliations:** 1 Division of Comprehensive Oral Health, Adams School of Dentistry, University of North Carolina, Chapel Hill, NC USA; 2 Division of Periodontology, Department of Stomatology, School of Dentistry, University of São Paulo, São Paulo, SP 05508-000 Brazil; 3 Department of Periodontics, School of Dental Medicine, University of Pennsylvania, Philadelphia, PA USA; 4 Division of Anatomy, Institute of Biomedical Sciences, University of São Paulo, São Paulo, SP 05508-000 Brazil; 5 Division of Periodontology, Dental School, University of Taubate, Taubaté, SP 12020-340 Brazil; 6 Department of Fundamental Chemistry, Institute of Chemistry, University of São Paulo, São Paulo, SP 05508-900 Brazil; 7 Division of Endodontics, Department of Dentistry, School of Dentistry, University of São Paulo, São Paulo, SP 05508-000 Brazil

**Keywords:** Drug discovery, Stem cells

## Abstract

Human periodontal ligament stem cells (PDLSCs) have been studied as a promising strategy in regenerative approaches. The protease-activated receptor 1 (PAR_1_) plays a key role in osteogenesis and has been shown to induce osteogenesis and increase bone formation in PDLSCs. However, little is known about its effects when activated in PDLSCs as a cell sheet construct and how it would impact bone formation as a graft in vivo. Here, PDLSCs were obtained from 3 patients. Groups were divided into control, osteogenic medium and osteogenic medium + PAR_1_ activation by TFLLR-NH2 peptide. Cell phenotype was determined by flow cytometry and immunofluorescence. Calcium deposition was quantified by Alizarin Red Staining. Cell sheet microstructure was analyzed through light, scanning electron microscopy and histology and transplanted to Balb/c nude mice. Immunohistochemistry for bone sialoprotein (BSP), integrin β1 and collagen type 1 and histological stains (H&E, Van Giesson, Masson’s Trichrome and Von Kossa) were performed on the ex-vivo mineralized tissue after 60 days of implantation in vivo*.* Ectopic bone formation was evaluated through micro-CT. PAR_1_ activation increased calcium deposition in vitro as well as BSP, collagen type 1 and integrin β1 protein expression and higher ectopic bone formation (micro-CT) in vivo.

## Introduction

Periodontal disease is a chronic inflammatory condition characterized by a dysbiotic microbiome mediated by host response patterns, which in advanced stages may result in a substantial breakdown of the tooth-supporting tissues, ultimately leading to tooth loss^1^. Periodontal tissue is a specialized distinctive structure composed by the alveolar bone, cementum and periodontal ligament. Damage to these structures severely compromises important biological functions, including bone remodeling, occlusal forces absorption and the masticatory function^1^.

Current both non-surgical and surgical treatments such as guided tissue regeneration (GTR) and guided bone regeneration (GBR) have been widely utilized in the clinical practice with the aim to eliminate the infection's primary cause and achieve regeneration, respectively. However, these treatment strategies have been shown to be ineffective in providing complete regeneration of hard and soft periodontal structures and the most common outcome is the formation of long junctional epithelium or the incomplete regeneration of the periodontal structures, and a long-term stable clinical regenerative outcome has not been yet achieved^2–4^.

Recently, several studies in the cell-based regenerative medicine field have been reported combining tissue engineering and stem cells^5–7^﻿. The use of cell sheets is a promising therapeutic strategy that allows the use of mesenchymal stem cells in a construct that maintains interconnected cells in contact with their extracellular matrix, providing stability and allowing delivery to possible therapies^8^. Cell sheets utilizing PDLSCs are well known to mimetize the natural periodontal ligament environment in a regenerative state and to enhance cellular signal communications that potentially stimulate regeneration of periodontal tissues^3^.

The protease-activated receptor type 1 (PAR_1_) is a G protein-coupled cell membrane receptor that regulates several intracellular signaling pathways related to enhancement of osteogenic differentiation, increase in mineralized matrix deposition and upregulation of cementogenic gene expression as well^9^. PAR_1_ activation plays a key role in osteogenesis and bone regeneration^5,10^ by enhancing osteoblast proliferation and differentiation^11^, increasing the synthesis of osteoprotegerin (OPG) in periodontal ligament cells and reducing the inflammatory osteoclastogenesis induced by LPS^12^. Furthermore, PAR_1_ has been shown to be associated with vascular and coagulatory processes^13^ as well as bone formation and osteoclasts differentiation impairment at bone healing early stages in an animal model^14^.

Whether PAR_1_ shows an effect on PDLSCs in a cell sheet in vivo model is still unknown. This present study focused on investigating PAR_1_ activation on the PDLSCs cell sheet outcome in an in vivo model.

## Results

### Periodontal ligament isolated cells present a diverse phenotypic population

**Figure 1:**
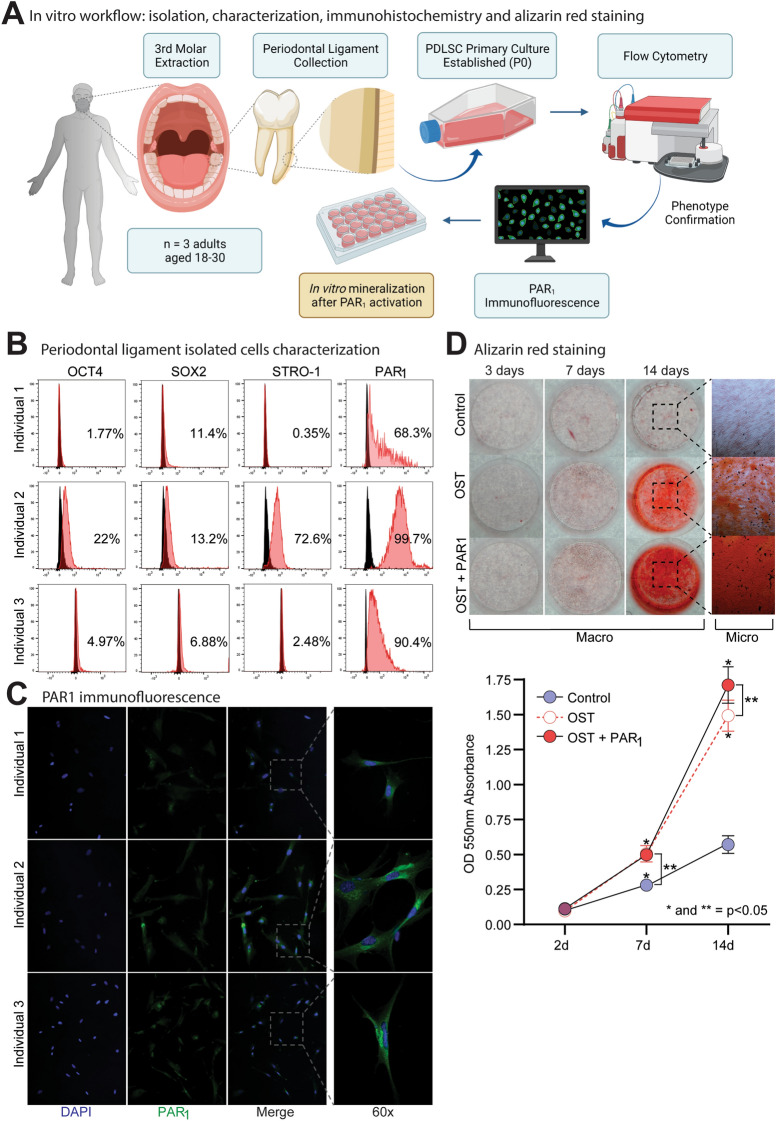
(**A**) Immunofluorescence, flow cytometry and alizarin red staining experiment flow. (**B**) Flow cytometry analysis showing the phenotypic characterization of PDLSCs for surface stemness, multipotent embryonic biomarkers (SOX2, OCT4, STRO-1) and PAR_1_ from three individuals (I1, I2 and I3). Unstained cells were used to set positive cell populations, *p* < 0.05. (**C**) PAR_1_ immunofluorescence expression in PDLSCs isolated cell lines. Images in 60X magnification. (**D**) Alizarin red staining for PDLSCs sheets (I2) after 14 days. Results are given as the mean ± SD. Figure (**A**) was created using BioRender.

We isolated PDLSCs from third molars from 3 individuals (I1, I2 and I3) and characterized the cell lines by flow cytometry and immunofluorescence for PAR_1_ (Fig. 1A). Although all cell lines were positive for pluripotency surface markers (Fig. 1C), only I2 presented a positive phenotypic profile for OCT-4 (22%), SOX2 (13.2%), and STRO-1 (72.6%) in agreement with the literature to be considered a PDLSC lineage (Fig. 1B and Supp. 1 A,B)^15^. Further, I2 presented a high percentage of PAR_1_ positive PDLSCs (~ 100%), as observed by Flow Cytometry and Immunofluorescence (Fig. 1B,C).

The Alizarin Red staining was carried out using only the I2 isolated mesenchymal stem cell line to evaluate whether the PAR_1_ activation played a role in their osteogenic differentiation and calcium deposition. Here, PAR_1_ significantly promoted an increase in calcium deposits through ARS assay in I2 PDLSCs (Fig. 1D). This way, we proceeded to perform further experiments using only the I2 cell line.

### Cell sheets characterization

**Figure 2:**
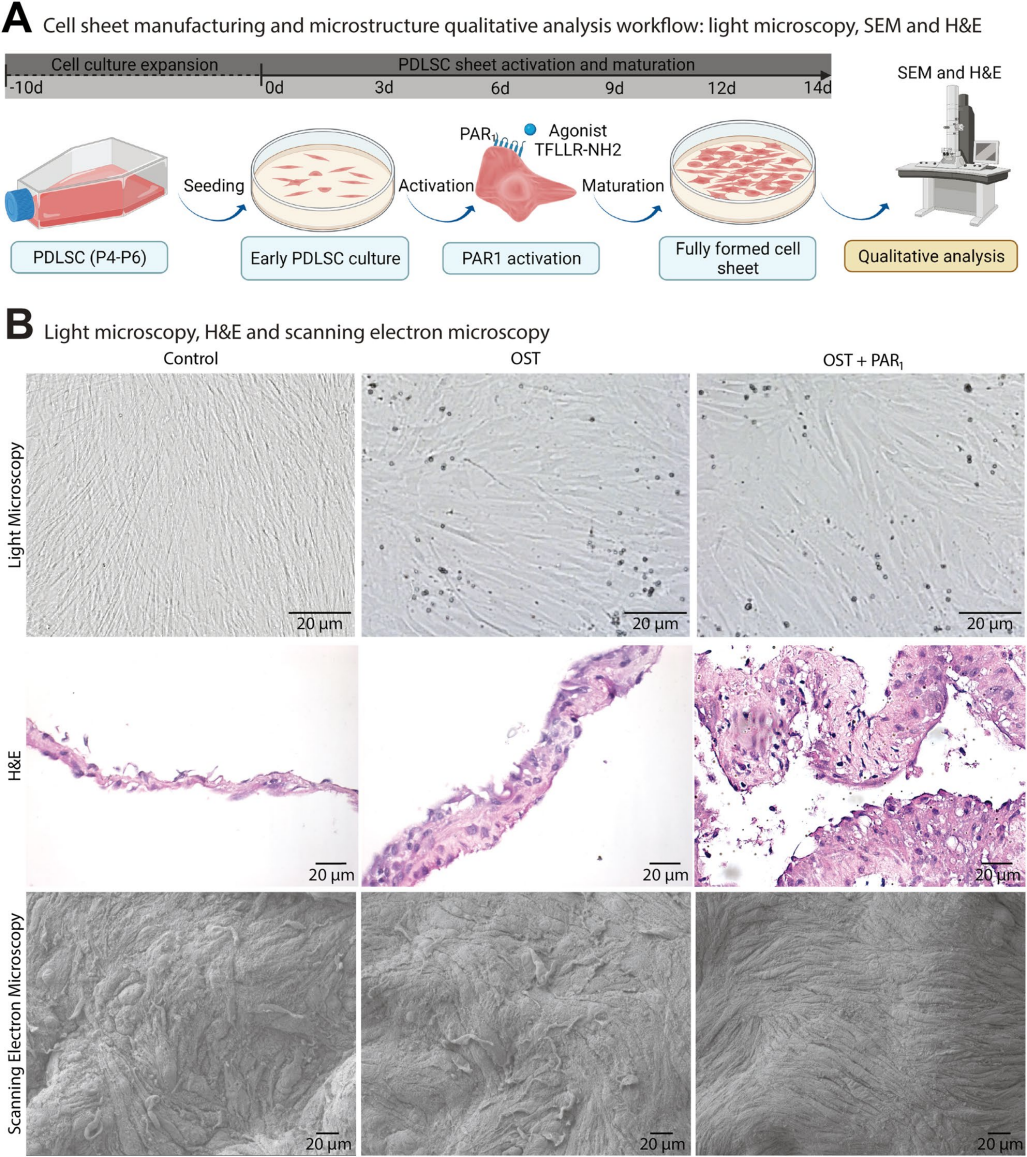
(**A**) Cell sheets manufacturing and macroscopic morphological characterization workflow. (**B**) Light microscopy (40x), cell sheet histological profile by H&E staining and microstructural analysis through scanning electron microscopy (400x) from cell sheets for the 3 experimental groups.

We characterized PDLSC sheets by scanning electron microscopy (SEM), H&E and direct light microscopy (Fig. 2A). After 14 days of culture with the three different groups, PDLSC sheets were fully formed and ready to undergo full detachment from the dishes (Supp. 2B). Light microscopy images showed a confluent fibroblastic-like profile as expected (Fig. 2B). Furthermore, qualitative analysis of the H&E sections detected differences in cell sheet thickness degree in the OST and OST + PAR_1_ groups when compared with the CTRL group (Fig. 2B).

### PAR_1_ activation in PDLSCs cell sheets enhanced mineralization after in vivo transplantation

**Figure 3:**
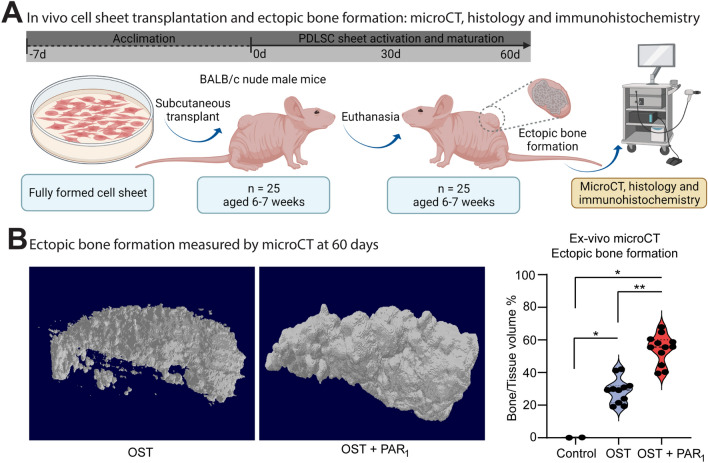
(**A**) Cell sheets in vivo transplant and ex-vivo analysis workflow. (**B**) Micro-CT analysis of ectopic bone formation in vivo after 60 days of transplantation (**B**) and the statistical analysis comparing the BV/TV% between the 3 groups CTRL, OST and OST + PAR_1_. (*) means a *p* < 0.05 compared to control. Results are given as the mean ± SD. Figure (**A**) was created using BioRender.

In order to evaluate the osteogenic potential of PAR_1_ activation in PDLSCs sheets, we performed in vivo experiments by transplanting cell sheets into the subcutaneous of Balb/c nude mice to evaluate the ectopic bone formation through micro-CT after a 60-days period (Fig. 3A). Micro-CT results were displayed in bone volume/tissue volume percentage (BV/TV %) (Fig. 3B). The OST + PAR_1_ group demonstrated a higher ectopic bone formation outcome in comparison with the other groups (*p* < 0.05) (Fig. 3B).

### PAR_1_ activation mediates osteogenesis in PDLSC sheets in vivo

**Figure 4:**
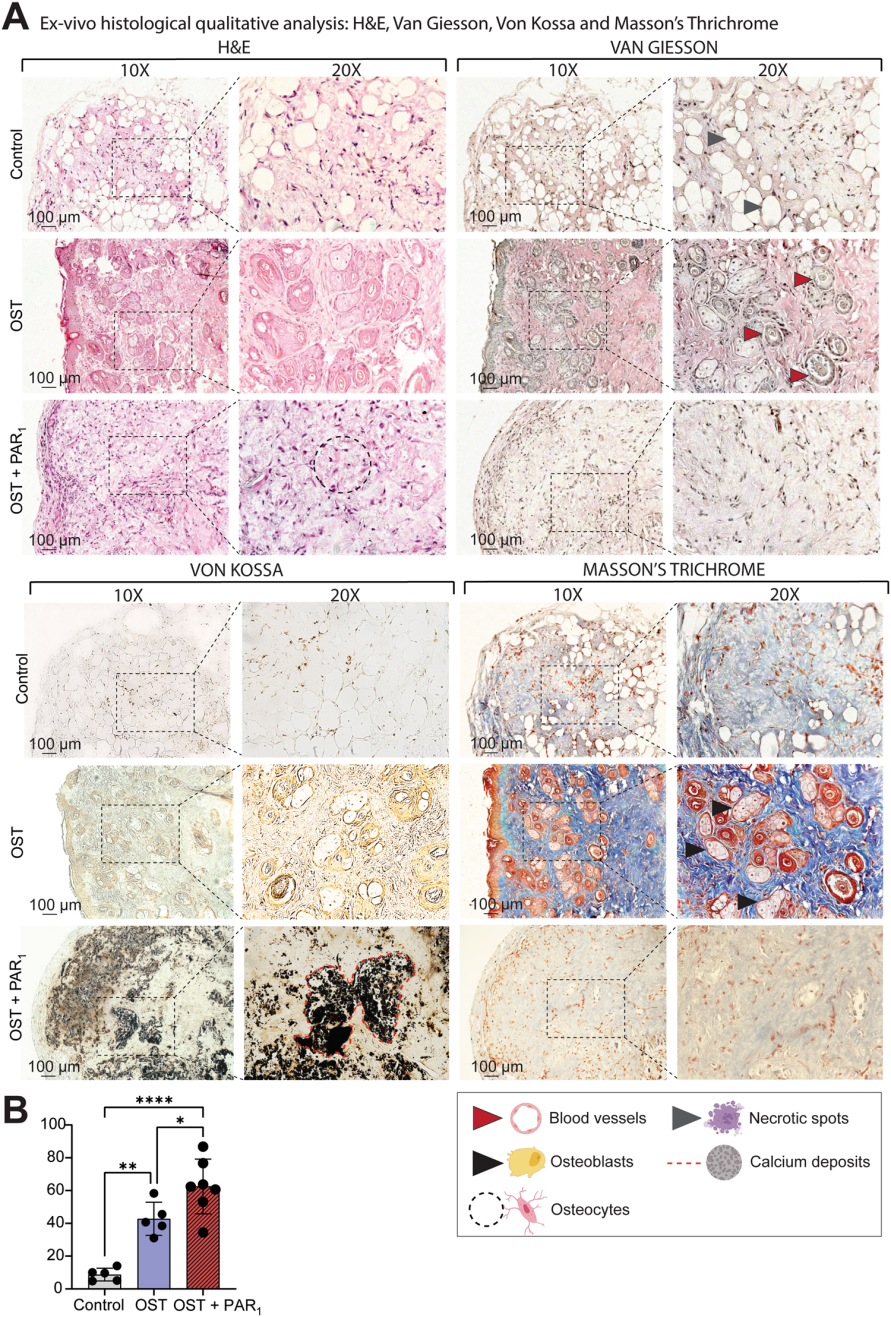
(**A**) Ex-vivo histological qualitative analysis from H&E, Van Gieson, Masson’s Trichrome and Von Kossa stains from the 3 groups after a 2-month transplantation period in balb/c nude mice. Images at 10x and 20x. CTRL group displayed connective tissue characteristics with necrotic spots (gray arrows) and no bone formation was detected in this group for the 4 stains. Osteoblast clusters (black solid arrows) and blood vessels (red solid arrows) were detected in higher abundance in the OST group when compared with the other groups. The OST + PAR_1_ group showed greater osteocyte numbers (black dotted circle) and higher calcium deposits (**B**) (red dotted line).

Histological qualitative analysis from H&E, Masson Trichrome, Van Gieson and Von Kossa stains from the 3 groups after a 2-month transplantation period in Balb/c nude mice suggested an increased osteogenic process displayed by greater presence of osteoblast clusters and lower abundance of undifferentiated fibroblast-like cells for the OST + PAR_1_ group (Fig. 4A) and increased calcium deposition in the neoformed bone matrix (Fig. 4B) when compared to the OST and CTRL groups (Fig. 4A,B). Furthermore, a qualitative analysis suggested a higher number of vessels in the OST group when compared to the other groups, suggesting that this group was in an intermediate osteogenesis stage when compared to the OST + PAR_1_ group (Fig. 4A).

### PAR_1_-induced mineralization in vivo is followed by an upregulation of osteogenic markers

**Figure 5:**
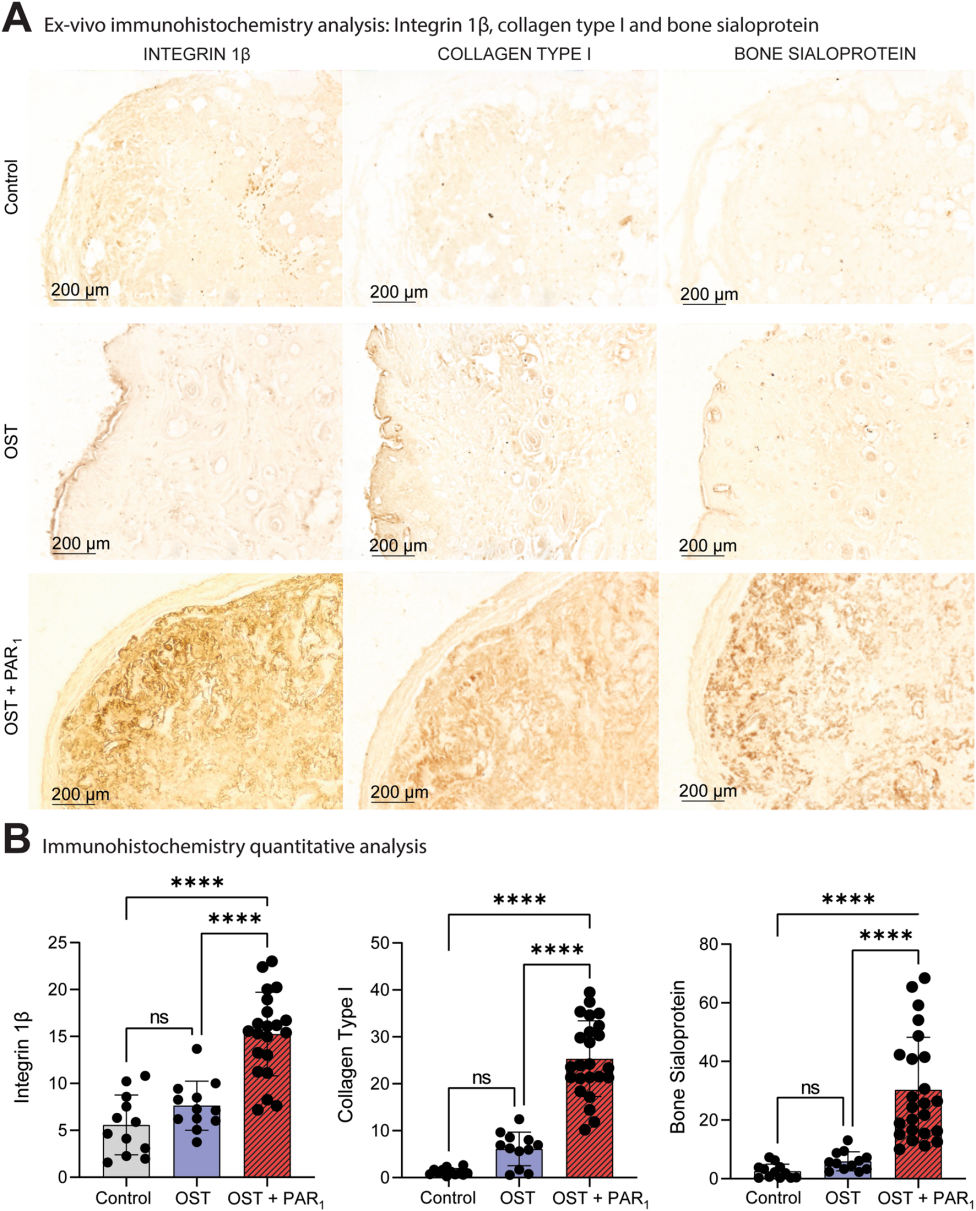
(**A**) Histological immunohistochemistry quantification analysis for collagen type I, integrin 1β and bone sialoprotein for the 3 groups *ex-vivo* samples after a 2-month transplantation period in balb/c nude mice. (**B**) A semi-quantification analysis was performed for the 3 protein markers and the PAR_1_ activation group showed greater staining for the 3 osteogenic protein markers after 60 days. Images at 10x. (*) *p* < 0.05. Results are given as the mean ± SD.

Immunohistochemistry results presented a higher positive expression of BSP in the OST + PAR_1_ group when compared with the other groups (Fig. 5A,B). Moreover, the expression of integrin 1β and collagen type 1 was detected in higher levels at the OST + PAR_1_ group when compared with the other groups (Fig. 5A,B).

## Discussion

In this study, we investigated the osteogenic potential of the PAR_1_ activation by using a cell sheet engineering strategy in an in vivo model. Previous studies have reported the role of PAR_1_ in the osteogenic processes demonstrating that its absence is associated with derived bone marrow cells migration impairment, an increase in osteoclast colonization, enhanced osteoclastogenesis and a decrease in mineralized bone deposition in vivo^14,16^.

On the other hand, PAR_1_ activation has been largely reported to upregulate bone formation by mediating proliferative responses in osteoblasts^5,10,14^. Our group has previously demonstrated that PAR_1_ activation in PDLSCs is specifically associated with an increase in mineralized nodule deposition, higher calcium concentration levels and increased PDLSC proliferation. In addition, PAR_1_ blockage decreased the calcium deposition, suggesting that PAR_1_ plays a major role in mineralization and differentiation of PDLSCs^17^.

In the present study, we isolated PDLSCs from three patients, characterized the surface marker phenotypes by flow cytometry and purposefully proceeded to evaluate the osteogenic differentiation potential in the cell line that presented a mesenchymal profile (I2). The non-specialized mesenchymal stem cells found in the periodontal ligament tissue, such as PDLSCs present pluripotency properties that allow differentiation into several cell types, including osteoblasts and cementoblasts. These cells exhibit a phenotype that is compatible with the positive expression of OCT4, STRO-1, CD-44 and CD-146 cell markers^18–22^. Further, cells with positive expression of STRO-1 were found to be related to periodontal tissue formation and expressed in dental follicle cells in mice^23,24^.

Our results demonstrated a possible correlation between the presence of stemness markers (OCT4, CD146, SOX2, CD-90, STRO-1, CD-44) and PAR_1_ prevalence, with a higher calcium deposition being found in I2 in the ARS essay, although further analysis is needed to confirm this association. At the same time, I2 was negative for CD14, CD31, and CD34, corroborating with PDLSCs phenotype present in the literature^15^.

Vascularization is a key mechanism in the earliest stages of osteogenesis during ectopic bone formation^25,26^ and angiogenesis is pivotal to enabling osteogenesis in vivo^27^. The absence of vascularization leads to tissue necrosis through hypoxia and nutrients dearth^28–30^. Cell sheets present improved vessel formation capability because the space between the cell layers allows for the formation of capillaries and, therefore, vascularization in newly transplanted grafts^31^.

In this study, we could identify qualitative histological differences between the groups regarding angiogenesis. The CTRL group exhibited a significant number of white spots suggesting necrotic areas, possibly due to a lack of vessel formation (Fig. 4A), especially when compared with the OST and OST + PAR_1_ groups (Fig. 4A). These findings could be explained by the absence of ascorbic acid in the CTRL group, in which depletion is related to angiogenesis impairment by inhibiting mature type IV collagen formation^32^. Further, the possible vascularization found in the OST group (Fig. 4A) may be explained since the group was at an earlier stage of osteogenesis when compared to the OST + PAR_1_ group, suggesting that the osteogenesis process may have been “accelerated” by PAR_1_ activation, which is also known to play a role in angiogenesis^33–35^ and coagulatory pathways^13^. These findings corroborate with several studies that demonstrated the pro-angiogenic role of PAR_1_ activation mainly through VEGF increased expression^33–35^. Further, VEGF has been shown to be an important angiogenic marker on studies evaluating the osteogenic and osteointegration processes on PDLSCs exposed to titanium-derived biomaterials *in vitro*^36,37^.

In fact, the enhanced osteogenic profile found in the OST + PAR_1_ group on the histological findings was consistent with the immunohistochemistry and micro-CT results in our study (Fig. 3). Evaluation of bone volume percentage (BV/TV %) through micro-CT displayed significantly higher values in the OST + PAR_1_ group when compared to the OST and CTRL groups (Fig. 3B).

In the bone neoformation processes, several extracellular molecules produced by differentiating osteogenic cells can be used to identify the stages of bone maturation in vivo^38^. Integrin 1β functions as a modulator, facilitating osteoblast attachment, spreading, adhesion, migration, and differentiation in osteogenesis via specific alpha(v)beta(3) signaling pathway^39,40^ and is capable of recognizing ligands with RGD-motifs, like BSP, which works as an osteoblast differentiation marker expressed during initial stages of bone formation^41,42^. Further, integrin 1β is capable of promoting angiogenesis by mediating human endothelial cell attachment and migration^40^ and is found to be able to integrate complexes that functions as a receptor to several collagen types^43^. In this matter, PAR_1_ activation is intrinsically related to an increase in collagen deposition in fibroblasts^44,45^ and, therefore, enhancing adhesion when integrin β1 is present in the substrate^46,47^.

In this study, the immunohistochemistry assay results for the OST + PAR_1_ group showed a higher protein expression for collagen type I, integrin 1β and BSP comparing with the other groups, evidencing an upregulated expression of important osteogenic immunological markers mediated by the PAR_1_ activation.

Finally, it is important to highlight some limitations present in this study. First, the research included only one donor cell line for the main experiments, which substantially reduces the external validity of the in vitro findings. Secondly, we found great difficulty in isolating mesenchymal strains from patients and a great difference in the surface markers characterization was found among the isolated cells. Moreover, although the flow cytometry data from the Individual 2 isolated cells were positive for a panel of surface and transcriptional factors of stemness, further differentiation into other mesenchymal cell types, i.e., adipocytes and chondrocytes, would be necessary to fully confirm their pluripotency phenotype. Yet, the osteoblastic differentiation verified in the present study suggests the therapeutic applicability in bone regeneration for PDLSCs presenting the herein characterized immunophenotyping profile. Finally, PAR_1_ expression was found to be widely variable, limiting the receptor activation associated outcomes for further PAR_1_ targeting therapies.

On the other hand, the use of cell sheets obtained through the removal of third molars seems to be a feasible option for further clinical therapies, since cell sheets present no associated adverse reactions because they can be obtained from and implanted in the same donor. Moreover, cell sheets are feasible to be used in regeneration procedures because the preservation of the extracellular matrix provides good structural stability and manageability, allowing this tissue engineering strategy to have a promising future as an option in regenerative treatments.

The main finding of this study is that the PAR_1_ activation in PDLSCs cell sheets enhanced osteogenesis in vivo*.* In addition, in vitro results from ARS reinforced that the presence of cell phenotype for stemness and PAR_1_ surface biomarkers are associated with an upregulation of osteogenesis in PDLSCs.

## Methods

We confirm that all methods were carried out in accordance with ARRIVE guidelines and relevant guidelines and regulations.

### Ethics Statement

Informed consent was obtained from all subjects and the ethical committee approval from the Ethics Committee Review Board at the School of Dentistry of the University of São Paulo (FO-USP-protocol number 029/2018) was obtained prior to the patient's teeth collection at the clinic of the School of Dentistry of the University of São Paulo (FO-USP). The utilization of Balb/c nude mice in this research was also approved by the Ethics Committee on Animal Use (CEUA) board at the Chemistry Institute of the University of Sao Paulo (IQ-USP) under the protocol number 98/2018.

### Cells isolation

Periodontal ligament stem cells were harvested from third molars from 3 systemically healthy individuals (I1, I2 and I3—18 to 30 years old). The inclusion criteria were: partially or totally erupted third molars and absence of periodontal disease.

Periodontal ligament tissue specimens were obtained through the scaling of the middle third of the root and PDLSCs were isolated using the explant technique^12,48^ in alpha-modified Eagle’s medium (α-MEM) (Thermo Fisher Scientific, Waltham, USA) supplemented with 10% fetal bovine serum (FBS) (Thermo Fisher Scientific, Waltham, USA), 100 μg/mL penicillin, 100 μg/mL streptomycin, and 0.5 mg/mL amphotericin B (Gibco, Invitrogen, Carlsbad, USA) at 37 °C in an atmosphere of 5% CO2 and 95% humidity^7^. After 14 days, cells from the explants achieved a 70% confluence degree and PDLSCs populations were used in passage 4 for all experiments.

### Pluripotency characterization

In order to identify the mesenchymal stem cell phenotype, approximately 5 × 10^5^ PDLSCs were incubated in 5% bovine serum albumin (BSA)/phosphate buffered saline (PBS) (Gibco, Invitrogen, Carlsbad, USA) 1 × at 4 °C in dark for 1 h with the following monoclonal antibodies: PAR_1_-FITC (Abcam, Cambridge, UK), OCT4-FITC (Abcam, Cambridge, UK), SOX2-FITC (Abcam, Cambridge, UK), STRO-1-FITC (Abcam, Cambridge, UK), CD14-FITC (eBioscience, San Diego, USA), CD90-FITC (eBioscience, San Diego, USA), CD31-PE (eBioscience, San Diego, USA), CD-44-PE (eBioscience, San Diego, USA), CD34-FITC (Biolegend, San Diego, USA) and CD146-PE (Biolegend, San Diego, USA) for 30 min at 4 °C. Unstained control was used to set gates. A total of 10–50,000 events were recorded and data analyzed through FlowJo (Becton Dickinson, Oregon, USA).

### Cell sheet culture and experimental design

PDLSCs at 1 × 10^6^ cells/cm2 were seeded in 100 mm plates for 24 h with α-MEM (Thermo Fisher Scientific, Waltham, USA) supplemented with 10% of FBS (Thermo Fisher Scientific, Waltham, USA) and 50 μg/mL vitamin C to induce cell sheet formation, as previously described^7^. Subsequently, cell sheets were assigned for one of the following experimental groups: (1) control medium (CTRL) composed of α-MEM (Thermo Fisher Scientific, Waltham, USA) supplemented with 10% of FBS (Thermo Fisher Scientific, Waltham, USA) and 50 μg/mL vitamin C (Sigma-Aldrich, St Louis, USA); (2) osteogenic medium (OST) (CTRL + 100 nM dexamethasone, 5 mM b-glycerophosphate and 50 µg/ml ascorbic acid (Sigma-Aldrich, St Louis, USA) and (3) osteogenic medium with the addition the PAR_1_ agonist peptide (100 nM TFLLR-NH2) (Tocris Bioscience Inc., Bristol, UK) (OST + PAR_1_). The culture medium was changed every 3 days for 14 days.

### Alizarin red staining

The PDLSCs from I2 were seeded in 6-well plates (5 × 10^4^ cells/cm^2^), in triplicate. After treatment with CTRL, OST and OST + PAR_1_ groups for 14 days, cell sheets were washed with 1 × PBS (Sigma-Aldrich, St Louis, USA), fixed with 4% paraformaldehyde (Sigma-Aldrich, St Louis, USA) for 15 min at room temperature (RT), washed with 1 × PBS (Sigma-Aldrich, St Louis, USA) again and incubated with a stirring solution of 2% Alizarin Red S (Sigma-Aldrich, St Louis, USA) in PBS (Sigma-Aldrich, St Louis, USA) (pH 4.2) (A5533) for 30 min at RT. For qualitative and macroscopic analysis, images were acquired using a microscope CDD camera (D7000, Nikon, Minato, Japan). For the quantitative analysis, 10% ammonium hydroxide (Sigma-Aldrich, St Louis, USA) solution was used to dilute calcium deposits and a spectrophotometer (Biotek, Winooski, USA) was used to measure the absorbance at 405 nm. ARS quantification was calculated using a standard-curve.

### Cell sheet characterization

#### Scanning electron microscopy

Cell sheets at 14-days treatment were fixed with Karnovsky's solution (Thermo Fisher Scientific, Waltham, USA) for 24 h. After this period, post-fixation was performed with osmium tetroxide (Sigma-Aldrich, St Louis, USA) for 2 h and dehydration, passing the material through an increasing series of alcohol (Sigma-Aldrich, St Louis, USA) from 70 to 100%. The samples were further dried under critical point conditions and sputter coated with gold. Observations were then performed by using a scanning electron microscope (S-4800; Hitachi, Tokyo, Japan).

#### Histological profile

Hematoxylin and eosin (Sigma-Aldrich, St Louis, USA) staining of cell sheets was also performed. Briefly, after detachment and fixation in 4% paraformaldehyde (Sigma-Aldrich, St Louis, USA) for 24 h, samples were paraffin embedded and sectioned in 4 µm thickness. After the sectioning process, the histological samples were placed in a water bath at 50 °C and captured to a histological slide and stored in an oven at 65 °C for 12 h to allow the paraffin remnants to be removed. Sequentially, paraffin removal dehydration protocol was completed using a sequence of xylol (Sigma-Aldrich, St Louis, USA) and alcohol (Sigma-Aldrich, St Louis, USA) solutions and the specimens proceeded a routine H&E staining following the manufacturer's guidelines.

### Ectopic bone formation assays

In vivo bone formation was evaluated using transplantation of cell sheets pre-incubated with the experimental groups described above. At 14 days of incubation, cell sheets were detached using a cell scraper (Corning, New York, USA), folded 4 times to acquire a cylinder shape and each 6-weeks old Balb/c nude mice received 2 dorsal transplants from the same experimental group, bilaterally. In total, 25 mice were distributed to the groups as follows: 11 (OST + PAR_1_), 11 (OST) and 3 (CTRL). After 60 days, mice were euthanized and the subcutaneous samples were removed and processed for the analysis described below.

### Computerized microtomography

After fixation, only the left side subcutaneous sample from each animal was scanned using a micro-CT system (Skyscan 1176, Bruker Biospin, Billerica, USA) with the following acquisition parameters: 45 kV, 8.71 μm resolution and 550 μA. Data obtained were processed, reconstructed into three-dimensional images using a reconstruction software (NRecon, Micro Photonics, Allentown, USA) and analyzed using a system software (CTAn, Bruker Biospin, Billerica, USA).

### Immunohistochemistry and histological analysis

Histological procedures were performed as described previously. H&E, Masson’s Trichrome, Van Gieson and Von Kossa stains (Sigma-Aldrich, St Louis, USA) were carried out following the manufacturer’s protocol. For immunohistochemistry analysis, after fixation in a 4% paraformaldehyde solution in PBS for 30 min, the samples were embedded in paraffin and sectioned at 20 µm. Cuts were subjected to dewaxing, rehydration, and permeabilized with 0.2% Triton X-100 (Sigma-Aldrich, St Louis, USA) for 10 min. Then, the sections were blocked in 3% hydrogen peroxide solution (Sigma-Aldrich, St Louis, USA) for 10 min followed by antigen retrieval with 10% citrate buffer (Sigma-Aldrich, St Louis, USA) for 15 min and blockage in 5% BSA (Sigma-Aldrich, St Louis, USA) for another 30 min. Then, exposure to the specific primary antibodies was performed for: β1 integrin—AB52971 (Abcam, Cambridge, UK), type I collagen—AB34710 (Abcam, Cambridge, UK), and BSP—AB8448 (Abcam, Cambridge, UK). The slides were then incubated with a secondary conjugated anti-rabbit IgG antibody—AB205718 (Abcam, Cambridge, UK) followed by an avidin-biotin complex (Abcam, Cambridge, UK) incubation for 30 min. The diaminobenzidine (DAB) solution (Abcam, Cambridge, UK) was applied every 2 min and the reaction was observed in a microscope. Specimens were dehydrated, cleared and mounted. Images from the specimens were obtained with a light microscope (E600, Nikon, Tokyo, Japan) and three images of each sample were acquired and quantified using Image J (NIH, Bethesda, USA).

### Statistical analysis

Experiments were performed in triplicate and data were expressed as means ± standard deviation (SD). Data were analyzed based on the comparison between the experimental group (OST + PAR_1_) with the osteogenic control group (OST) and the control (CTRL). One-way ANOVA followed with post-hoc Tukey’s test was used. A significance *P value* of 0.05 was established for all tests and the data analysis was performed (GraphPad Prism™ Version 6.0c, La Jolla, USA).

## Supplementary Information

Supplementary Information.

## Data Availability

The datasets used and/or analysed during the current study available from the corresponding author on reasonable request.
