# Supplementary material for: Osteogenesis in human periodontal ligament stem cell sheets is enhanced by the protease-activated receptor 1 (PAR1) in vivo
Source: Sci Rep. 2022 Sep 18;12:15637. doi: 10.1038/s41598-022-19520-x (PMC9482923; doi:10.1038/s41598-022-19520-x)
Supplement: Supplementary file 1 — Supplementary Information. [file 41598_2022_19520_MOESM1_ESM.pdf]

Supplementary Figure 1

A PDLSC flow cytometry gating strategy for the unstained control

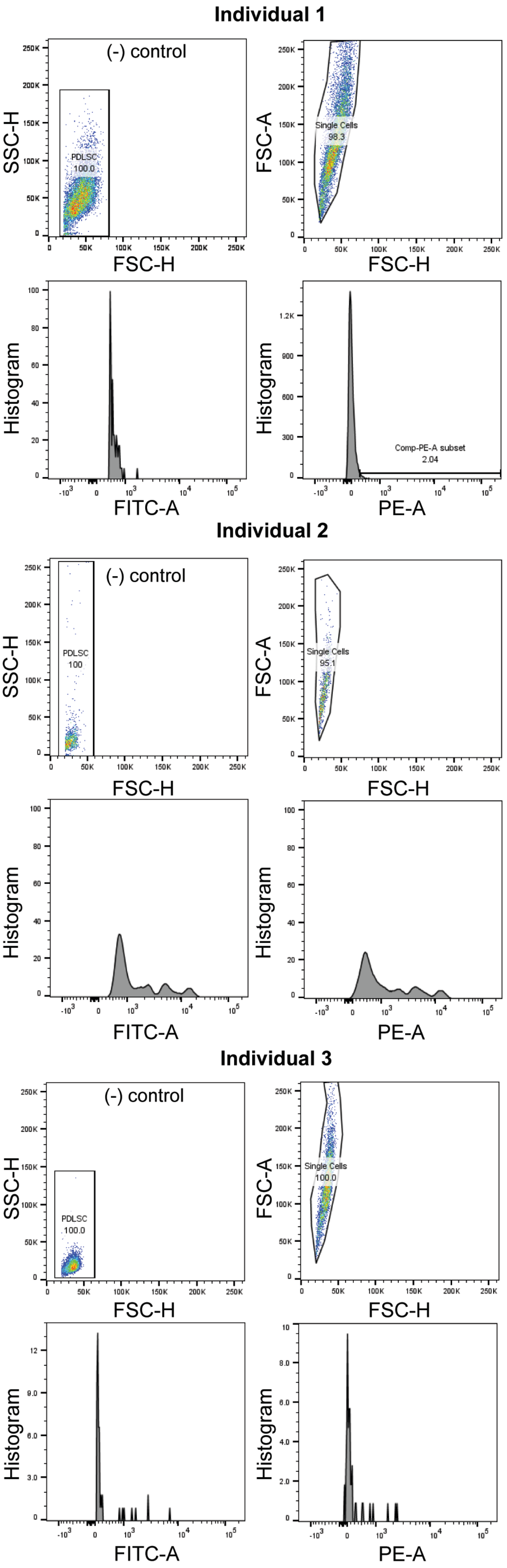

B Additional flow cytometry characterization

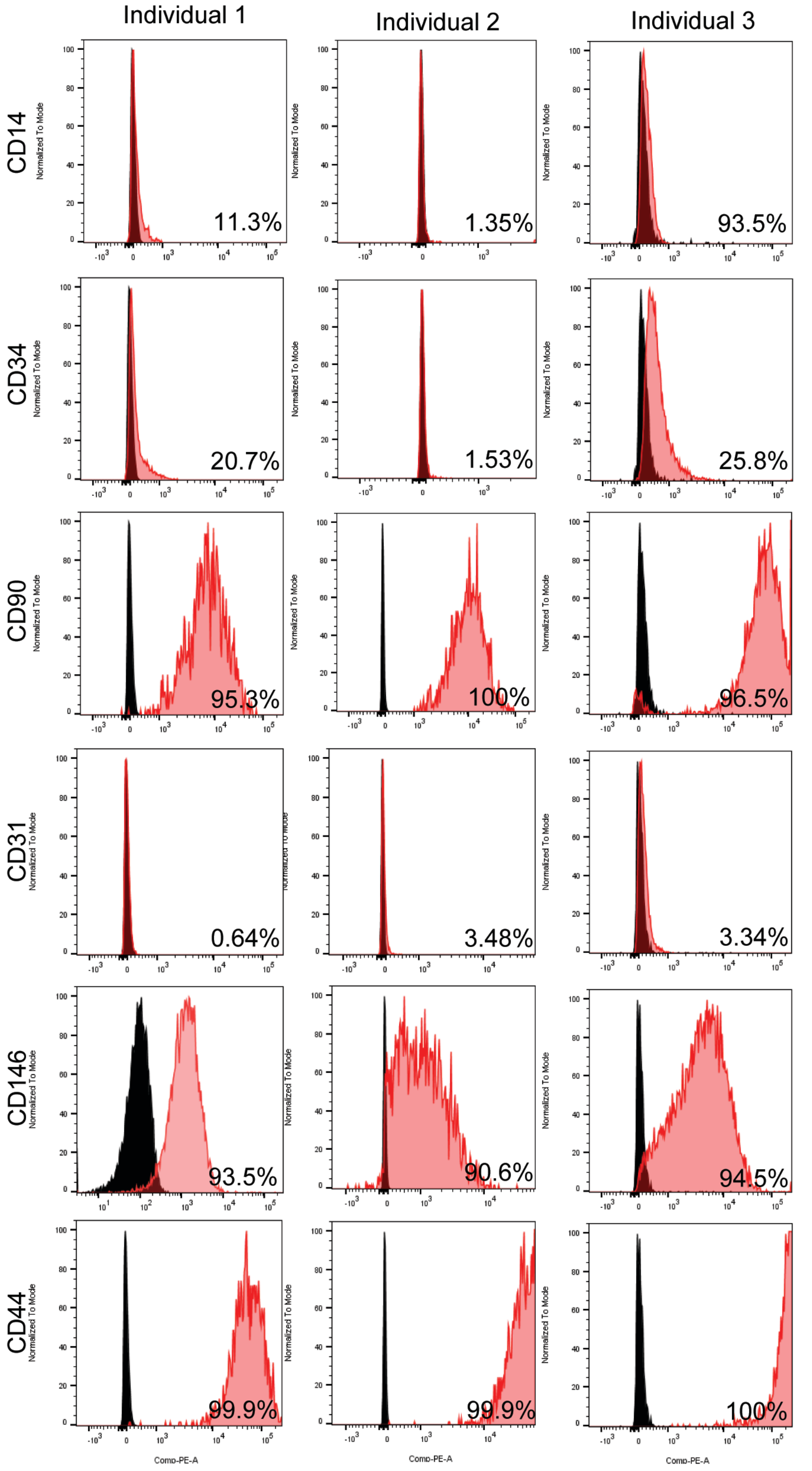

Legends: (A) Gating strategy for the unstained control used in the flow cytometry for the three isolated cell lines. **(B)** Additional flow cytometry markers used to characterize the phenotype from the isolated cell lines.

Supplementary Figure 2

A Primary cell isolation through the explant technique

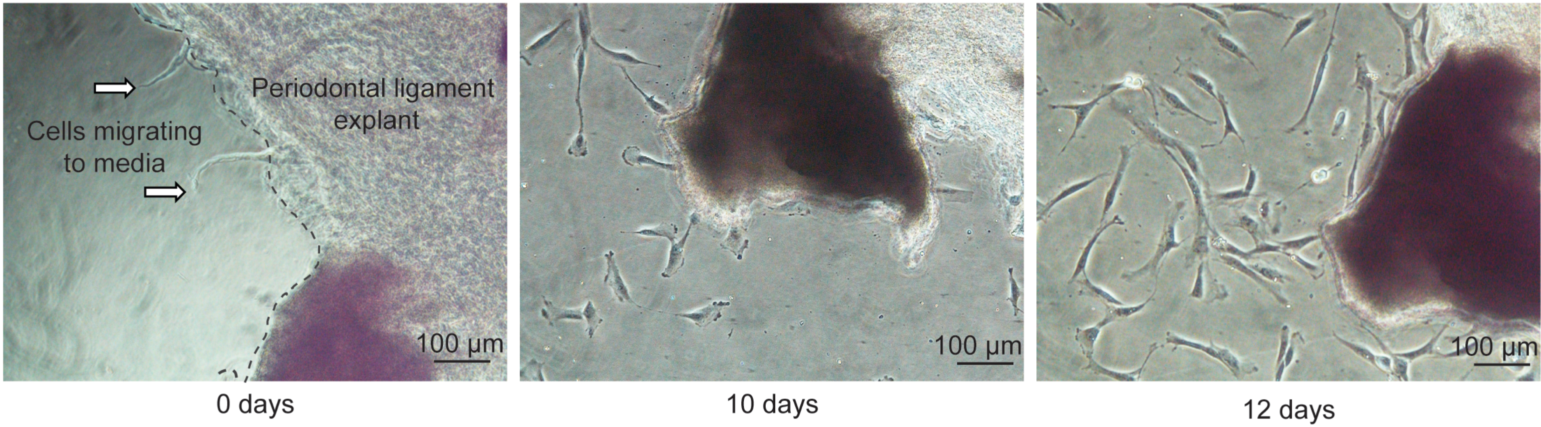

B Cell sheet dettachment, folding and grafting workflow

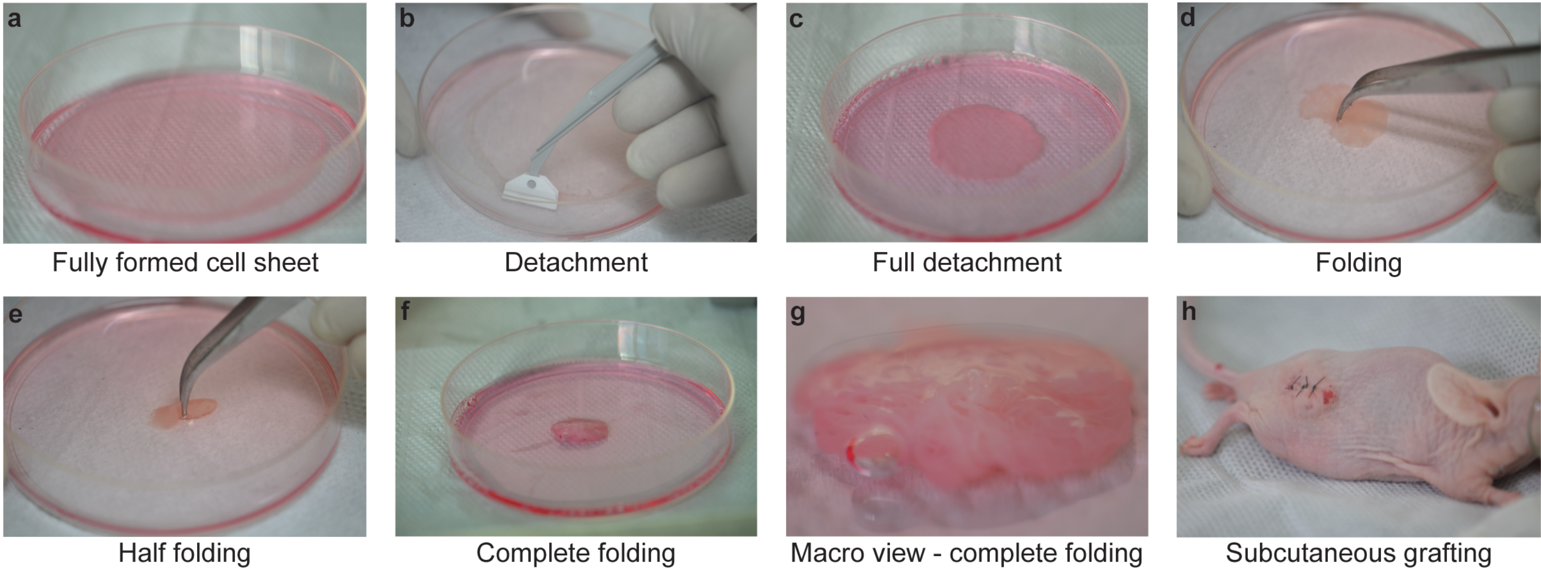

Legends: (A) Primary cell culture establishment (P0) and growth after the periodontal ligament explant was acquired for 12 days. **(B)** After 14 days of culture, cell sheets were detached, folded and transplanted bilaterally into the subcutaneous of Balb/c nude mice (2 per mice).
